# Effect of Extract and Synthesized Derivatives of Isolated Compound from *Symplocos chinensis* f. Pilosa Ohwi on Neuropathic Pain in Mice

**DOI:** 10.3390/molecules26061639

**Published:** 2021-03-15

**Authors:** Hyun-Yong Kim, Soo-Hyun Park, Guanglei Zuo, Kang Hyuk Kim, Seung Hwan Hwang, Hong-Won Suh, Soon-Sung Lim

**Affiliations:** 1 Department of Food Science and Nutrition, Hallym University, 1 Hallymdeahak-gil, Chuncheon 24252, Korea; khy9514@nate.com (H.-Y.K.); guangleizuo@foxmail.com (G.Z.); whs528@naver.com (K.H.K.); isohsh@gmail.com (S.H.H.); 2 R&D Center, Frontbio Inc., 32 Soyanggang-ro, Chuncheon 24232, Gangwon-do, Korea; shyun1017@hanmail.net; 3 R&D Center, Huons Co., Ltd., 55 Hanyangdaehak-ro, Ansan 15588, Gyeonggi-do, Korea; 4 Department of Pharmacology, College of Medicine, Hallym University, Hallymdeahak-gil, Chuncheon 24252, Korea; hwsuh34@gmail.com; 5 Institute of Korean Nutrition, Hallym University, 1 Hallymdeahak-gil, Chuncheon 24252, Korea; 6 Institute of Natural Medicine, Hallym University, 1 Hallymdeahak-gil, Chuncheon 24252, Korea

**Keywords:** *Symplocos chinensis* f. pilosa Ohwi, neuropathic pain, antinociceptive effect, synthesized derivatives, 5-succinoxymethylfurfural

## Abstract

Neuropathic pain is described as the “most terrible of all tortures that a nerve wound may inflict.” The aim of the present study was to demonstrate the antinociceptive effect of *Symplocos chinensis* f. pilosa Ohwi water extract (SCW) and synthesized derivatives of the isolated compound. The antinociceptive effect was tested using the acetic acid-induced writhing and 5% formalin tests. Antinociceptive effects on neuropathic pain were evaluated using the von Frey test with chronic constriction injury (CCI) and surgical nerve injury (SNI) models and tail-flick test with a vincristine-induced pain model. An Ames test was also conducted. 5-hydroxymethylfurfural (5-HMF) was isolated and derivatives were synthesized with various acid groups. Among the plant water extracts, SCW showed significantly effective activity. Additionally, SCW presented antinociceptive effects in the neuropathic pain models. The SCW water fraction resulted in fewer writhes than the other fractions, and isolated 5-HMF was identified as an effective compound. Because 5-HMF revealed a positive response in the Ames test, derivatives were synthesized. Among the synthesized derivations, 5-succinoxymethylfurfural (5-SMF) showed the best effect in the neuropathic pain model. Our data suggest that SCW and the synthesized compound, 5-SMF, possess effective antinociceptive activity against neuropathic pain.

## 1. Introduction

Compounds derived from natural plants have served as a source of medicine throughout human history [1]. From 1981 to 2007, approximately half of all new drugs were based on natural plant-derived sources [2]. Natural plants provide an extensive reservoir of compounds with important structural diversity and offer chemical entities for medicine [1].

Pain is a psychological, negative affective response accompanied by complex behavioral changes to alleviate discomfort [3]. Pain can be divided into neuropathic pain, cause by disease, and inflammatory pain (nociceptive pain), which arises from stimuli from damaged tissue [4]. Non-neuropathic pain begins with the disease of non-neural tissues [5], whereas neuropathic pain is caused by a primary insult to the central or peripheral nervous system and is classified into neuralgias of peripheral nerves or central neuropathic pain [6]. Many natural extracts and compounds exert effects against neuropathic pain, including *Cannabis sativa* [7], *Rosmarinus officinalis* L. [8], *Olea europaea* L. [9], capnellene [10] and resveratrol [11]. 

Considerable research on *Symplocos* has been directed to their diverse biological activities, including their anti-human immunodeficiency virus activity, inhibitory activities against phosphodiesterase, and antitumor applications [12]. Plants in this genus are also used to treat inflammation and remedy for menstrual disorders [13] and some of the chemical constituents of the *Symplocos* genus have been isolated, including triterpenoids, flavonoids, lignans, phenols, steroids, alkaloids, and iridoids [14]. One species in this genus, *Symplocos chinensis*, is a herb found in southern China and is used as a folk medicine to treat disease, such as malaria, tumefaction, and nephritis [15]. Furthermore, terpenoid saponins extracted from this plant show antitumor activity [16].

The current mainstream of therapy for neuropathic pain is based on the use of antiepileptics, such as gabapentin or carbamazepine, over a long period; however, little is known about the safety and efficacy of these drugs [17]. Therefore, the aim of this study was to discover synthetic compounds based on 5-hydroxymethylfurfural (5-HMF) isolated from *S. chinensis f*. pilosa Ohwi extract for the treatment of neuropathic pain. Because 5-HMF has a possible toxic mechanism [18], we used a *Salmonella typhimurium* mutagenicity based test (Ames test), which is a suitable bacterial assay to detect mutagenic potential [19].

The aim of the present study was to identify effective resources for the treatment of neuropathic pain from *S. chinensis* f. pilosa Ohwi extract, isolated compounds, and synthetic compounds using a mouse model of neuropathic pain to discover functional food resources.

## 2. Results

### 2.1. Antinociceptive Effect of Various Plant Water Extracts in Acetic Acid-Induced Writhing Test

**Figure 1:**
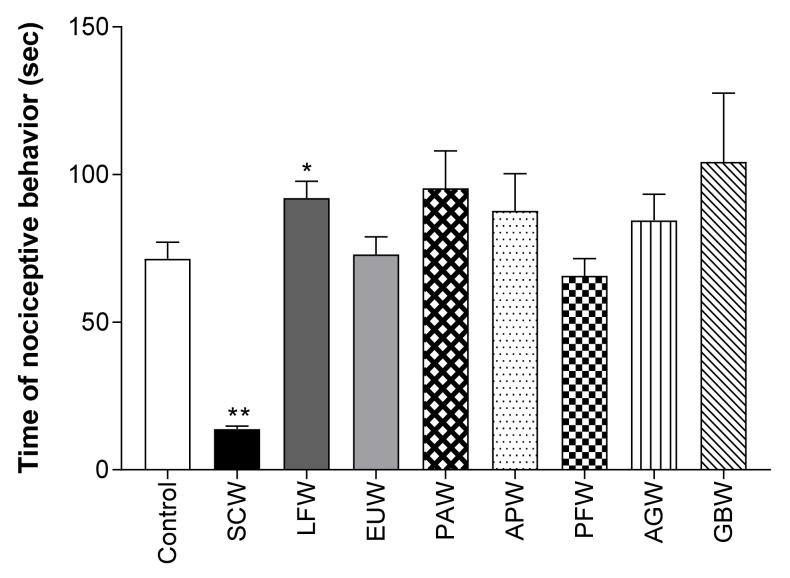
Antinociceptive activity of water extract from various plants in the acetic acid induced abdominal writhing test. Data are expressed as means ± SD; *n* = 8 mice per group (* *p* < 0.01, ** *p* < 0.001 vs. control group). SCW: *Symplocos chinensis* f. pilosa Ohwi water extract, LFW: *Liquidambar formosana* Hance water extract, EUW: *Eucommia ulmoides* water extract, PAW: *Phellodendron amurense* water extract, APW: *Agrimonia pilosa* Ledeb water extract, PFW: *Perilla frutescens* var. crispa water extract, AGW: *Angelica gigas* water extract, GBW: *Ginkgo biloba* water extract.

To screen the most effective extract in the pain model, we used 100 different plant water extracts (complete data not shown). Among the extracts, *Symplocos chinensis* f. pilosa Ohwi water extract (SCW) showed the highest antinociceptive effect at 100 mg/kg (b.w., p.o.) (Figure 1). Therefore, SCW was selected for subsequent experiments.

### 2.2. Antinociceptive Effect of SCW in Acetic Acid-Induced Writhing Test

**Figure 2:**
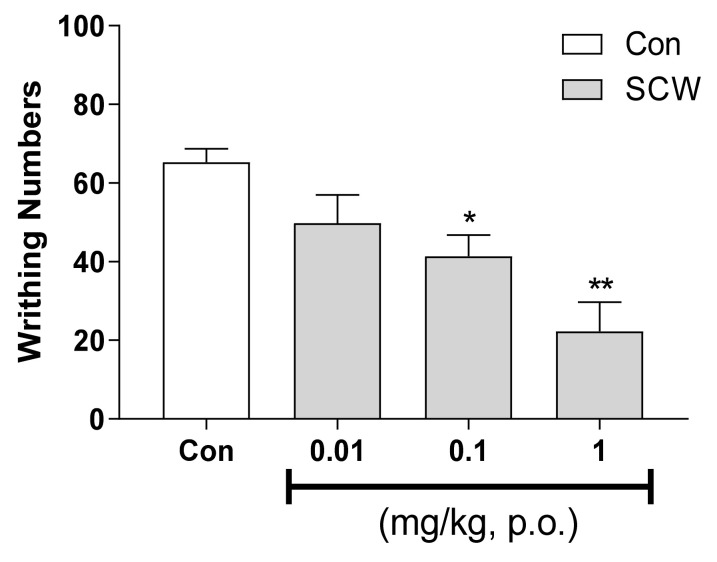
Effect of *Symplocos chinensis* f. pilosa Ohwi water extract at various concentrations in the acetic acid-induced abdominal writhing test. Data are expressed as mean ± SD; *n* = 8 mice per group. (* *p* < 0.01, ** *p* < 0.001 vs. control group).

As the effect was shown in SCW, the efficacy of various concentrations of SCW was confirmed. SCW showed dose-dependent effects at 0.01, 0.1 and 1 mg/kg (b.w., p.o.) (Figure 2). Significantly fewer writhes were observed in the SCW-treated group than in the control group.

### 2.3. Antinociceptive Effect of SCW on 5% Formalin-Induced Nociception

**Figure 3:**
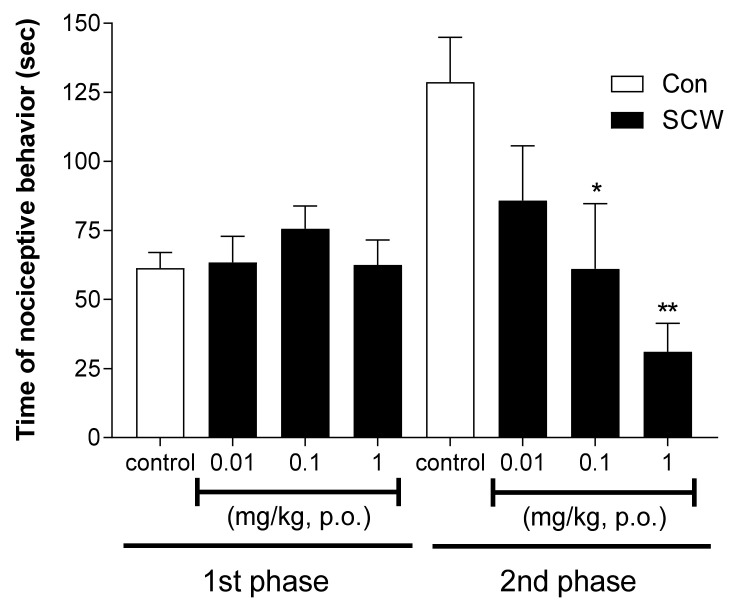
Effect of *Symplocos chinensis* f. pilosa Ohwi water extract (SCW) in the formalin test in mice. Data are expressed as mean ± SD; *n* = 8 mice per group. (* *p* < 0.05, ** *p* < 0.001 vs. control group).

Since peripheral pain was confirmed using acetic acid induced writhing test, a formalin test was processed to confirm the effect of SCW in central pain. In the formalin-induced nociception model, SCW did not show a significant different effect compared with the control group in the first phase (Figure 3). However, in the second phase, the SCW-treated group presented effectively reduced nociceptive behavior in a dose-dependent manner (0.01, 0.1 and 1 mg/kg [b.w., p.o.]). At doses of 0.1 and 1 mg/kg SCW, the nociceptive behavior time was significantly lower than that in the control group.

### 2.4. Antinociceptive Effect of SCW on Mechanical Pain Thresholds in CCI and SNI Neuropathic Pain Model

**Figure 4:**
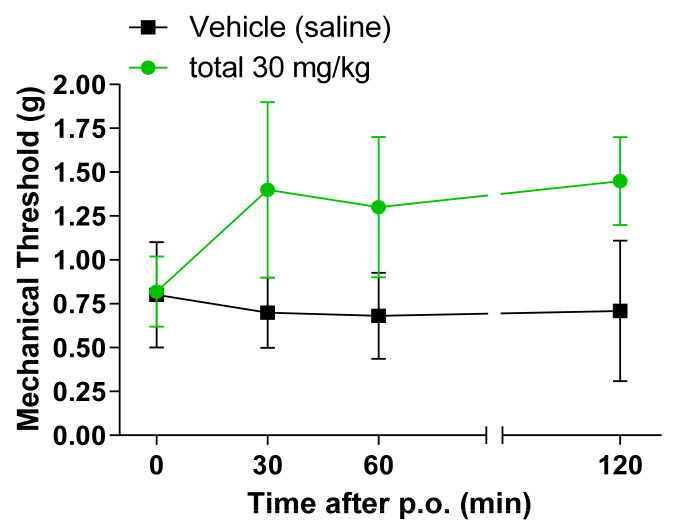
Effect of *Symplocos chinensis* f. pilosa Ohwi water extract in the Chronic Constriction Injury (CCI) von Frey test in mice. Data are expressed as mean ± SD; *n* = 4 mice per group.

**Figure 5:**
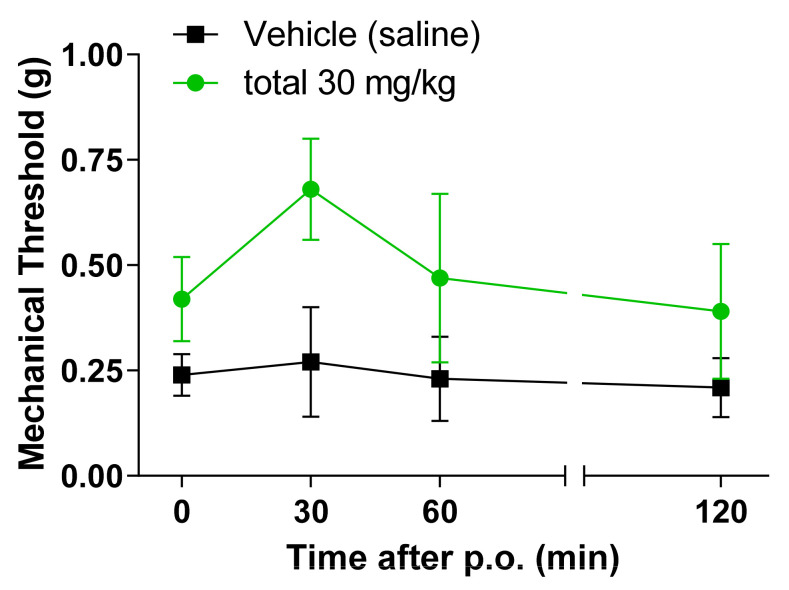
Effect of *Symplocos chinensis* f. pilosa Ohwi water extract in the Spared Nerve Injury (SNI) von Frey test in mice. Data are expressed as mean ± SD; *n* = 4 mice per group.

An experiment was conducted to confirm the effect of SCW on neuropathic pain, and CCI and SNI model were applied for the experiment. The SCW-treated group showed potent activity as an effective antinociceptive extract (Figure 4 and Figure 5). The 30 mg/kg SCW group presented an increased mechanical threshold from 30 min to 120 min in the CCI model after treatment. In the SNI model, the SCW-treated group showed an increased mechanical threshold at 30 min, but this decreased with time. However, the mechanical threshold number in the SCW-treated group was higher than that in the vehicle group at all the times (0, 30, 60, and 120 min). 

### 2.5. Antinociceptive Effect of SCW on the Tail-Flick Test in Vincristine Induced Pain Model

**Figure 6:**
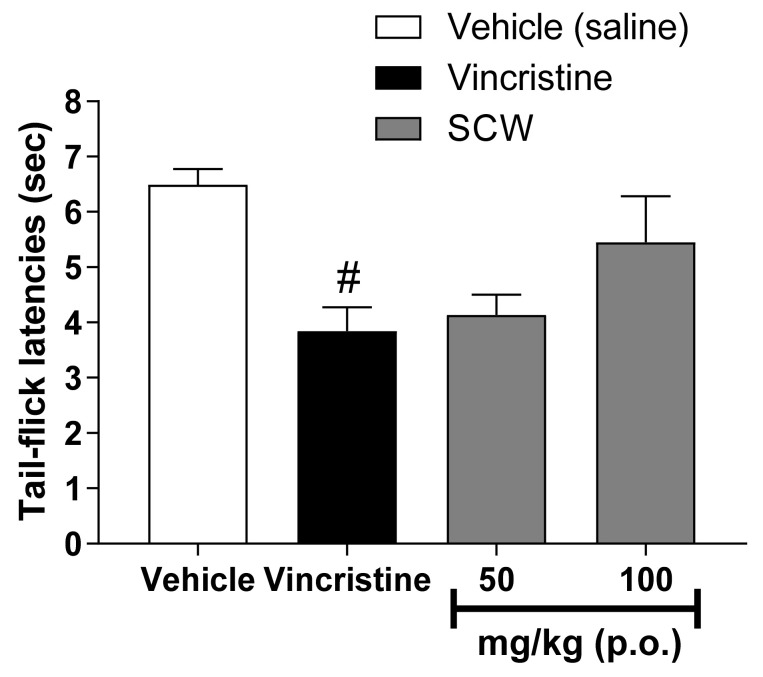
Effect of *Symplocos chinensis* f. pilosa Ohwi water extract in the vicrisitine-induced pain model. Data are expressed as mean ± SD; *n* = 6 mice per group. (^#^ *p* < 0.001 vs. vehicle group).

To confirm the analgesic effect of SCW itself, a tail flick test was performed using a vincristine induced pain model. SCW at 50 and 100 mg/kg was used in the tail-flicking test in a vincristine-induced pain model (Figure 6). The vincristine-treated group showed a lower latency time than the vehicle group. The SCW-treated group presented higher latency times than the vincristine-treated group, but the difference was not significant. 

### 2.6. Antinociceptive Effect of SCW Fractions in Acetic Acid-Induced Writhing Test

**Figure 7:**
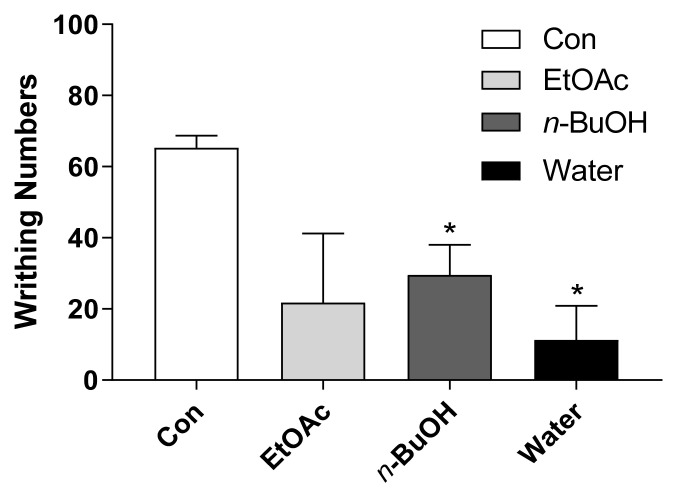
Effect of *Symplocos chinensis* f. pilosa Ohwi water extract (SCW) solvent fractions in the acetic acid-induced abdominal writhing test. Data are expressed as mean ± SD; *n* = 4 mice per group. (* *p* < 0.001 vs. control group).

Based on the antinociceptive effect of SCW, SCW solvent fractions were used to confirm the activity (200 mg/kg, b.w., p.o.). Three fractions from SCW were used: the EtOAc, *n*-BuOH, and water. Among them, *n*-BuOH and water fractions resulted in significantly fewer writhes than the control treatment, and the water fraction resulted in fewer writhes than the *n*-BuOH fraction (Figure 7). Therefore, the SCW water fraction (SCWW) was used for effective compound isolation.

### 2.7. Antinociceptive Effect of Isolated Sub-Fractions from SCWW in Acetic Acid-Induced Writhing Test

**Figure 8:**
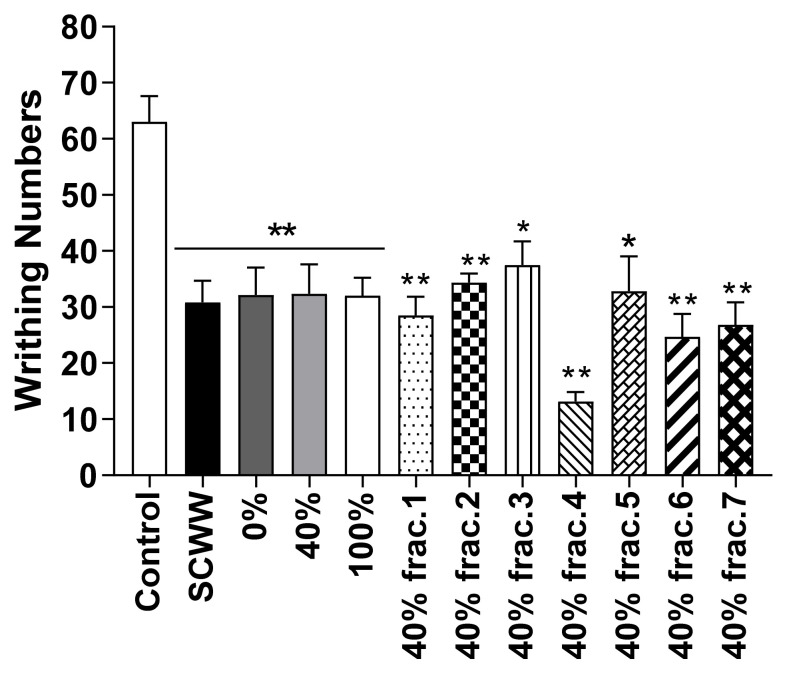
Effect of isolated sub-fractions from water layer of *Symplocos chinensis* f. pilosa Ohwi water extract in the acetic acid-induced abdominal writhing test. Data are expressed as mean ± SD; *n* = 6 mice per group. (* *p* < 0.01, ** *p* < 0.001 vs. control group).

**Figure 9:**
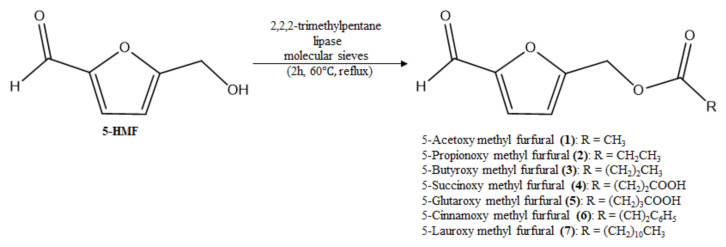
Isolated 5-hydroxymethylfurfural (5-HMF) (HPLC data, NMR spectra and EI-MS data in Supplementary Materials) from *Symplocos chinensis* f. pilosa Ohwi water extract (SCW) and synthesized derivatives.

For the isolation of the active compound from SCWW, a Diaion HP-20 column was used with 0, 40, and 100% MeOH. All MeOH isolated fractions (10 mg/kg, b.w., p.o.) showed significantly fewer writhes than the control group. Among them, the active compound was isolated from the 40% MeOH isolated fraction (Figure 8). Seven fractions were collected from the 40% MeOH isolated fraction, and fraction 4 presented the highest antinocicpetive effect among the fractions (10 mg/kg, b.w., p.o.). Fraction 4 was confirmed to be 5-HMF (Figure 9) and a positive mutagenic response was observed with 5-HMF in the Ames test (data not shown). Therefore, we synthesized derivatives of 5-HMF to discover more effective compounds without a mutagenic response. All derivatives showed negative mutagenic responses in the Ames test.

### 2.8. Antinociceptive Effect of Isolated 5-HMF and Derivatives in Acetic Acid-Induced Writhing Test and on 5% Formalin-Induced Nociception

**Figure 10:**
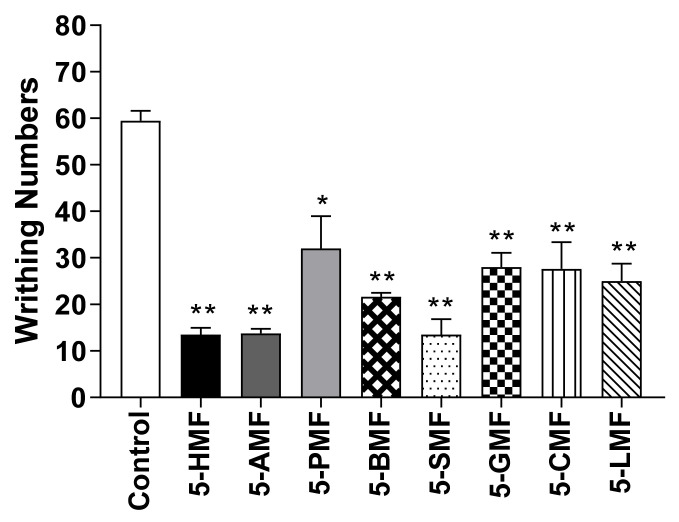
Effect of 5-HMF and its derivatives in the acetic acid-induced abdominal writhing test. Data are expressed as mean ± SD; *n* = 8 mice per group. (* *p* < 0.01, ** *p* < 0.001 vs. control group). 5-HMF: 5-hydroxy methyl furfural, 5-AMF: 5-acetoxy methyl furfural, 5-PMF: 5-propionoxy methyl furfural, 5-BMF: 5-butyroxy methyl furfural, 5-SMF: 5-succinoxy methyl furfural, 5-GMF: 5-glutaroxy methyl furfural, 5-CMF: 5-cinnamoxy methyl furfural, 5-LMF: 5-lauroxy methyl furfural.

**Figure 11:**
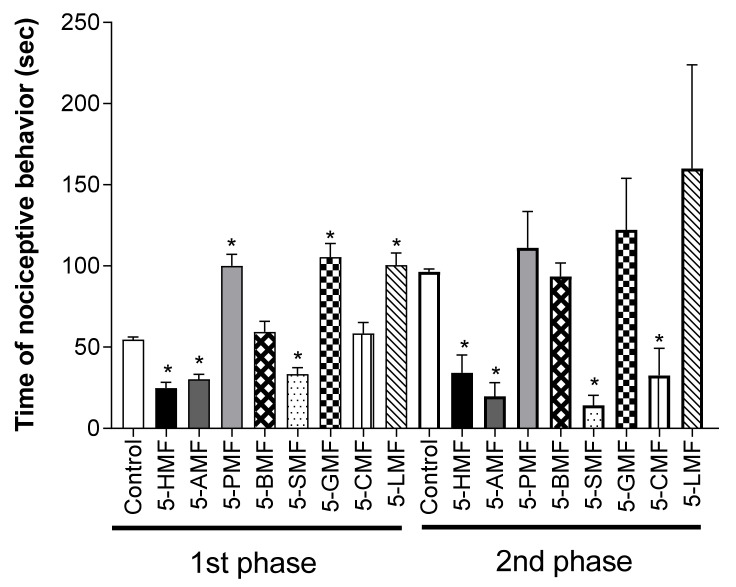
Effect of 5-HMF and its derivatives in the formalin test in mice. Data are expressed as mean ± SD; *n* = 8 mice per group. (* *p* < 0.001 vs. control group). 5-HMF: 5-hydroxy methyl furfural, 5-AMF: 5-acetoxy methyl furfural, 5-PMF: 5-propionoxy methyl furfural, 5-BMF: 5-butyroxy methyl furfural, 5-SMF: 5-succinoxy methyl furfural, 5-GMF: 5-glutaroxy methyl furfural, 5-CMF: 5-cinnamoxy methyl furfural, 5-LMF: 5-lauroxy methyl furfural.

To confirm the effect of 5-HMF and its derivatives, a 10 mg/kg dose of compound was used in the acetic acid-induced writhing test and 5% formalin-induced nociception model. 5-HMF and its derivatives resulted in significantly fewer writhes than the control treatment, among which 5-SMF showed the highest antinociceptive effect (Figure 10). Furthermore, in the formalin-induced nociception model, the 5-SMF-treated group showed a significantly lower nociceptive behavior time at all phases (*p* < 0.001) than the control group (Figure 11).

### 2.9. Antinociceptive Effect of 5-SMF and Gabapentin in Acetic Acid-Induced Writhing Test 

**Figure 12:**
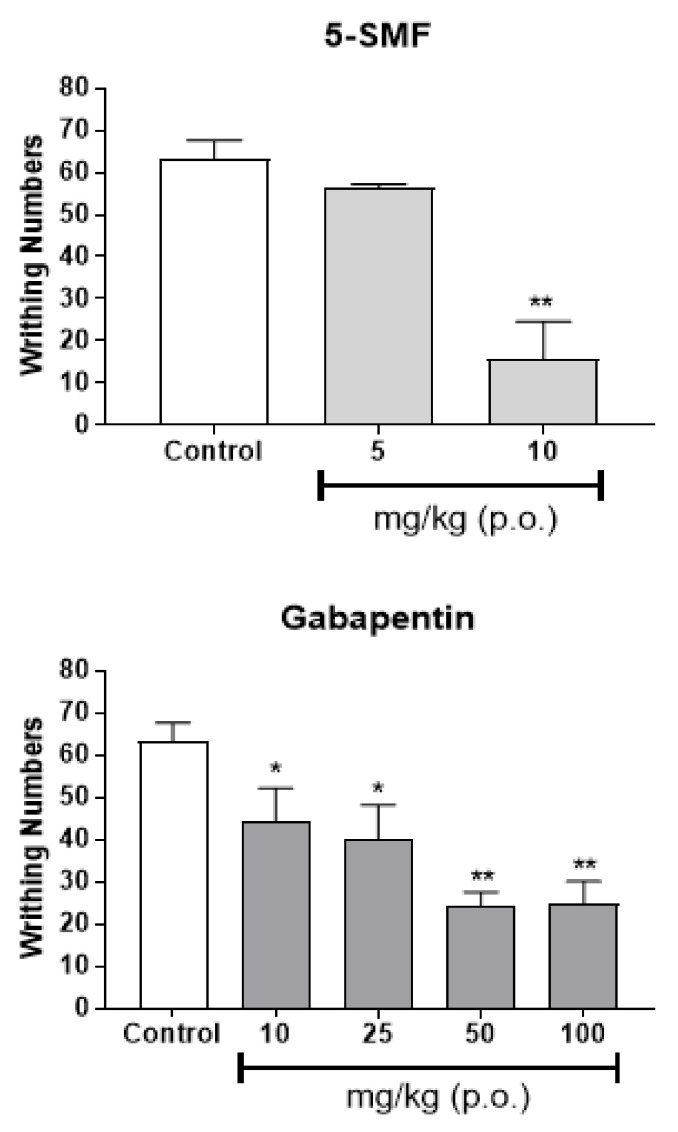
Effect of 5-SMF and gabapentin in the acetic acid-induced abdominal writhing test. Data are expressed as mean ± SD; *n* = 4 mice per group. (* *p* < 0.05, ** *p* < 0.001 vs. control group).

**Figure 13:**
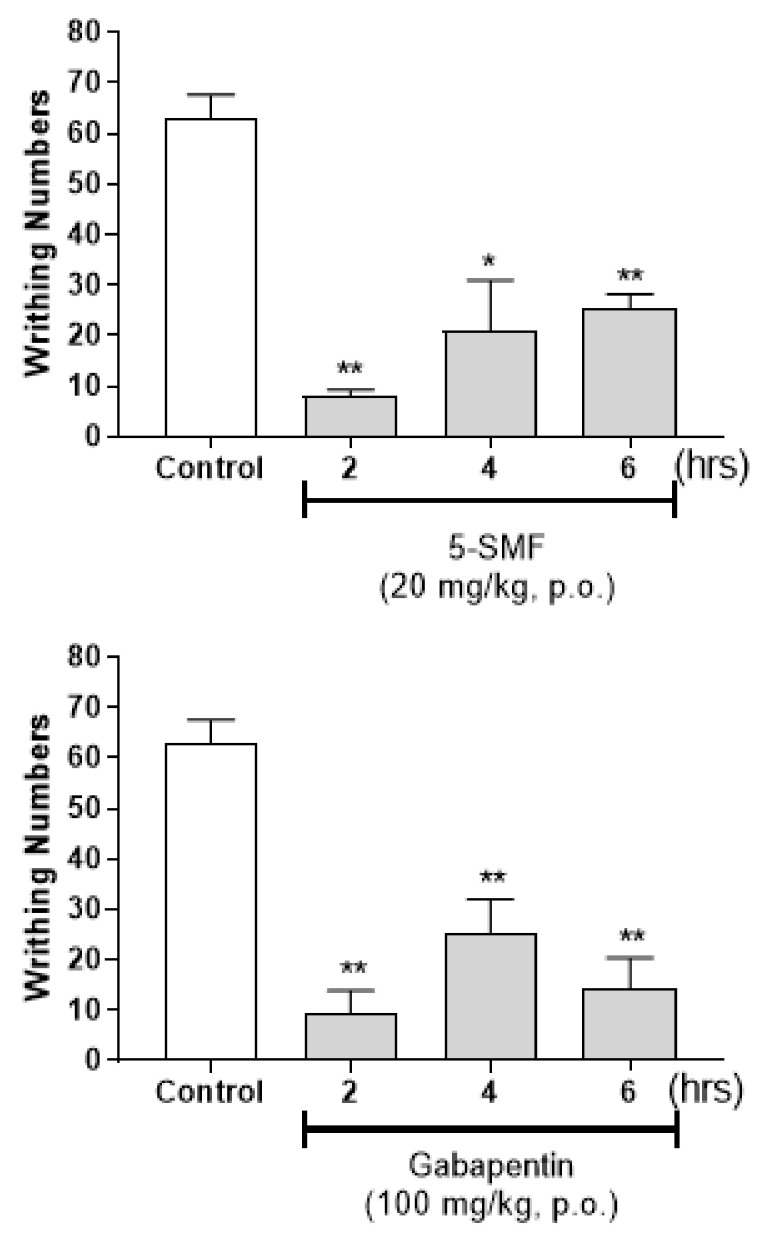
Time-dependent effect of 5-SMF and gabapentin in the acetic acid-induced abdominal writhing test. Data are expressed as mean ± SD; *n* = 4 mice per group. (* *p* < 0.01, ** *p* < 0.001 vs. control group).

As effect of 5-SMF was confirmed among the 5-HMF based synthetic products, experiments were conducted to confirm the antinociceptive effects of 5-SMF. The antinociceptive effect of 5-SMF (5 and 10 mg/kg, b.w., p.o.) was compared with that of gabapentin (10, 25, 50 and 100 mg/kg, b.w., p.o.), the most commonly used drug for neuropathic pain, using the acetic acid-induced writhing test. 5-SMF presented a dose-dependent antinociceptive effect at a lower concentration than gabapentin (Figure 12). Additionally, 5-SMF (20 mg/kg, b.w., p.o.) resulted in fewer writhes than the control treatment 2, 4 and 6 h after administration (* *p* < 0.01, ** *p* < 0.001 vs. control group) (Figure 13). Gabapentin (100 mg/kg, b.w., p.o.) also resulted in fewer writhes than the control group during the same period. These results suggest that 5-SMF has a potential antinociceptive effect at lower doses than gabapentin.

### 2.10. Antinociceptive Effect of 5-SMF and Gabapentin on 5% Formalin-Induced Nociception 

**Figure 14:**
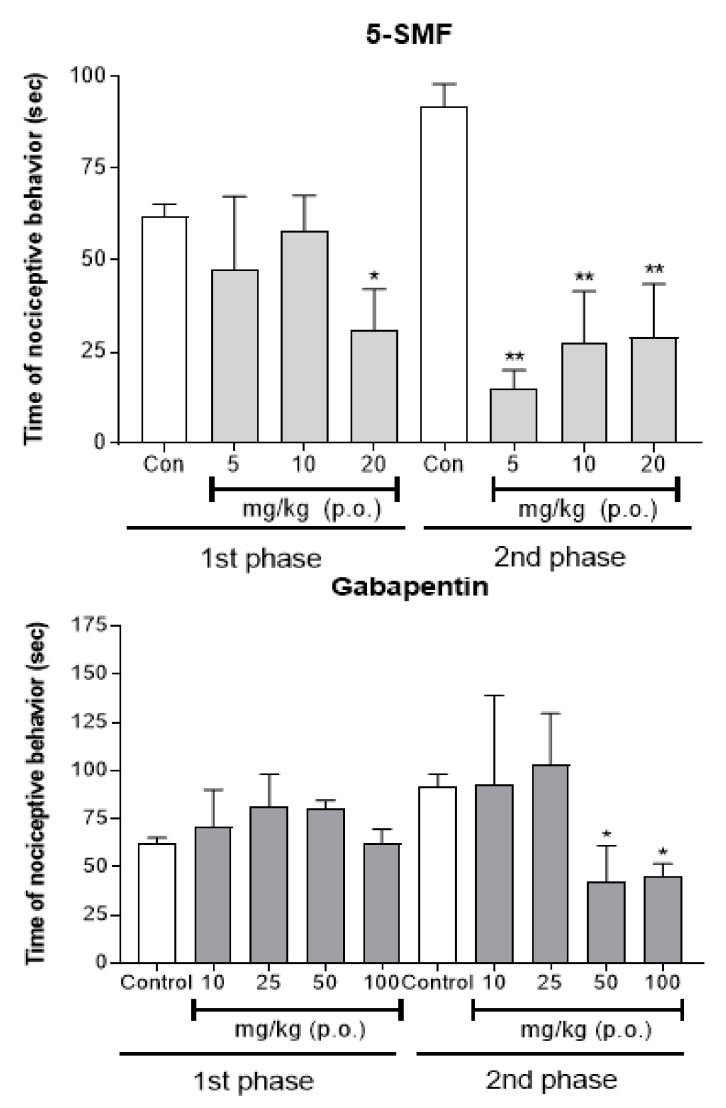
Effect of 5-SMF and gabapentin in the formalin test in mice. Data are expressed as mean ± SD; *n* = 4 mice per group. (* *p* < 0.01, ** *p* < 0.001 vs. control group).

In the formalin-induced nociception model, the antinociceptive effect of 5-SMF (5, 10 and 20 mg/kg, b.w., p.o.) was compared with that of gabapentin (10, 25, 50, and 100 mg/kg, b.w., p.o.) (Figure 14). The 5-SMF-treated group presented significantly less nociceptive behavior than the control group at a dose of 20 mg/kg in the 1st phase, and all doses in the 2nd phase (* *p* < 0.01, ** *p* < 0.001 vs. control group). The gabapentin-treated group did not show any effect compared with the control group in the 1st phase, but significantly less nociceptive behavior was detected at doses of 50 and 100 mg/kg in the 2nd phase. 

### 2.11. Antinociceptive Effect of 5-SMF in Tail-Flick and Hot-Plate Tests

**Figure 15:**
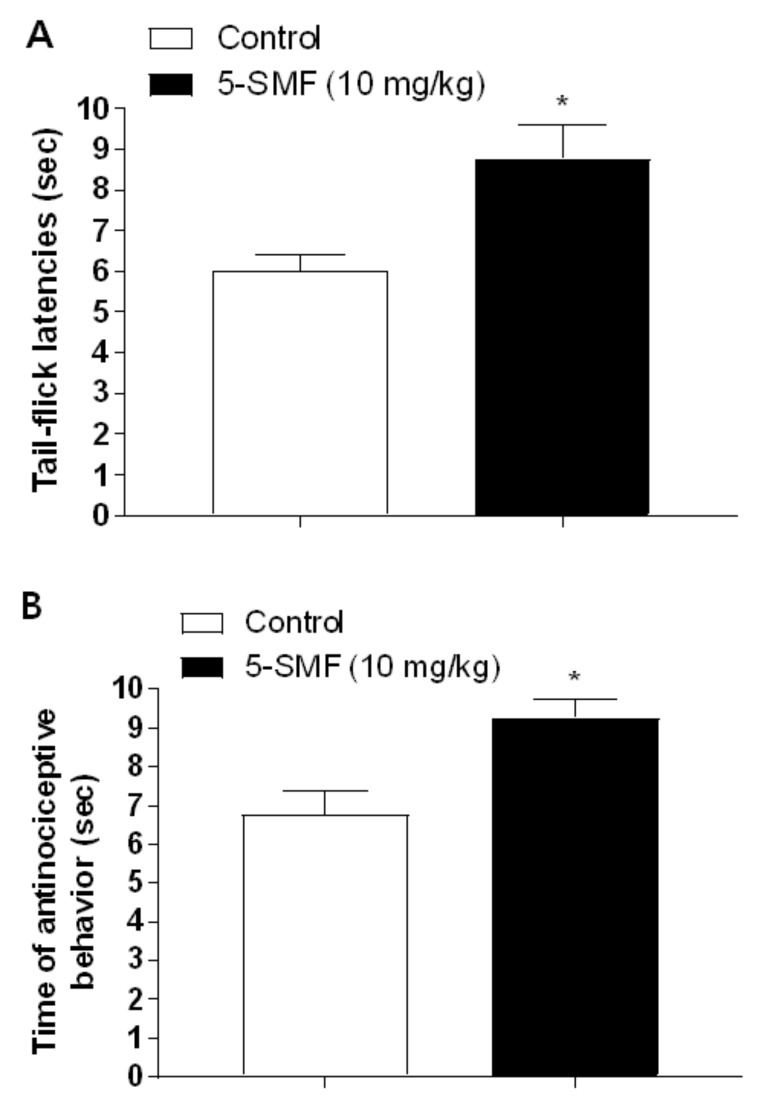
Effect of 5-SMF and gabapentin in the tail-flick test (**A**) and hot-plate tests (**B**). Data are expressed as mean ± SD; *n* = 4 mice per group (* *p* < 0.05 vs. control group).

The results presented in Figure 15 showed the effect of 5-SMF at 10 mg/kg (b.w., p.o.) using tail-flick and hot-plate tests. In the tail-flick test, the 5-SMF-treated group showed a significantly higher latency time than the control group (Figure 15A). In the hot-plate test, the 5-SMF-treated group presented more antinociceptive behavior than the control group (Figure 15B).

### 2.12. Antinociceptive Effect of 5-SMF and Gabapentin in the Tail-Flick Test in Vincristine-Induced Pain Model

**Figure 16:**
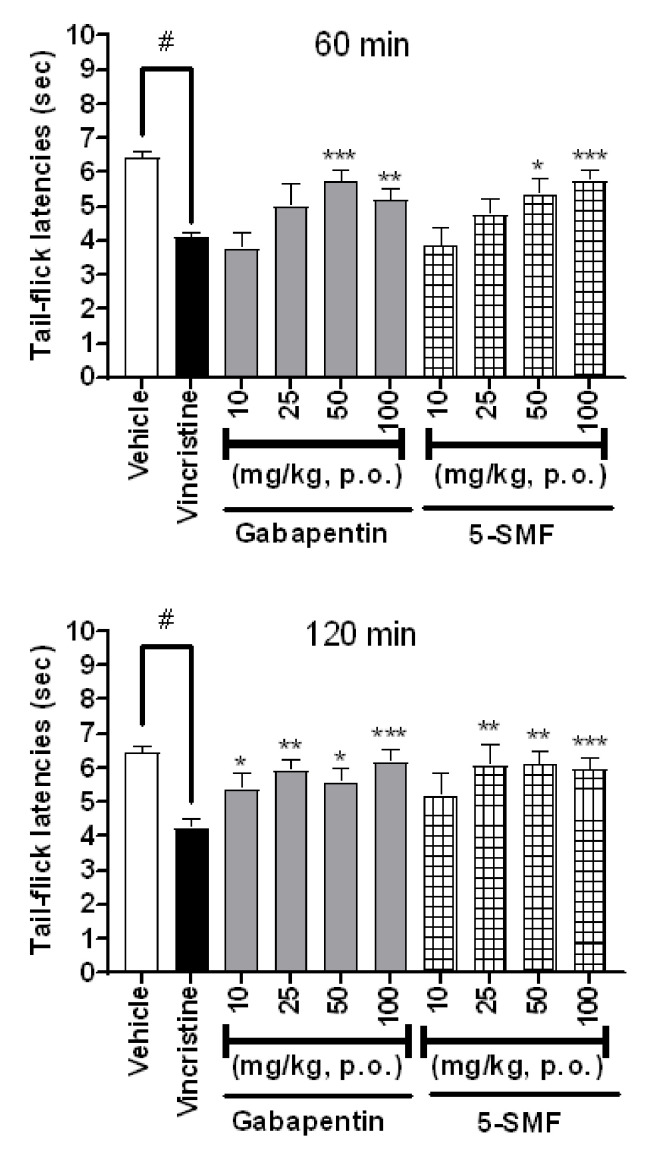
Effect of 5-SMF and gabapentin in the vincristine-induced pain model at 60 and 120 min. Data are expressed as mean ± SD; *n* = 7 mice per group. (^#^ *p* < 0.001 vs. vehicle group, * *p* < 0.05, ** *p* < 0.01 and *** *p* < 0.001 vs. vincristine group).

The results presented in Figure 16 show the effects of 5-SMF and gabapentin at doses of 10, 25, 50, and 100 mg/kg (b.w., p.o.) using a tail-flicking test in a vincristine-induced neuropathic pain model. Compared with the vincristine group, the 5-SMF-treated group showed a dose-dependent increase in latency time 60 min after sample treatment, and the 50 and 100 mg/kg-treated groups showed significantly increased latency time in both the 5-SMF and gabapentin groups. After 120 min of treatment, both the 5-SMF- and gabapentin-treated groups presented significantly higher latency times than the vincristine-treated group at dose of 25, 50 and 100 mg/kg. 

## 3. Discussion

In the present study, the results indicate that SCW and the synthesized compound, 5-SMF, show antinociceptive effects in a neuropathic pain model. 

There are various methods to evaluate the antinociceptive effect of an extract, among which, the acetic acid-induced writhing test model’s visceral pain to evaluate peripheral antinociceptive activity [20]. Another method, the formalin-induced pain test, was used to evaluate neurogenic pain. The first phase (neurogenic pain) reflects the direct chemical stimulation of C-fibers, and the second phase (inflammatory pain) represents peripheral tissue inflammation [21]. Our data, regarding the effect of SCW in an acetic acid-induced writhing test and a formalin-induced pain test (2nd phase) suggest that SCW has antinociceptive activity on peripheral pain.

After evaluating the effect of the SCW using two pain tests, we used CCI and SNI models to determine the effect of SCW on neuropathic pain. Neuropathic pain is a debilitating condition that results from partial injury to a peripheral nerve [22] and is described as “the most terrible of all tortures which a nerve wound may inflict” [23]. The CCI model is one of the frequently used model for the study of neuropathic pain and its treatment [24], and the SNI model is a useful tool for the study of nerve injury-induced neuropathic pain [22]. The SCW showed a higher effect in the CCI and SNI models than the vehicle group. These results suggest that the SCW may act to reduce neuropathic pain. 

The tail-flicking test is simple and commonly used to study analgesic efficiency [25] and measure spinal nociceptive response latency [26]. To model neuropathic pain, mice were treated with vincristine. Vincristine is one of the most common drugs for the treatment of malignancies, and it induces painful peripheral neuropathy as a side effect [27]. When vincristine was administered to the mice, they showed significantly fewer tail-flicks than the vehicle group. Thus, a vincristine-induced neuropathic pain model was confirmed. When 50 and 100 mg/kg doses of SCW were administered to each mouse, the results showed the dose-dependent but non-significant effect of SCW on neuropathic pain (*p* > 0.05). These results were analyzed to confirm the possibility of the effect of SCW on neuropathic pain. 

After evaluating the effect of SCW concentrations, we investigated the effect of solvent fractions and isolated the effective compound from the SCW sample. When the acetic acid-induced pain model was treated with SCW solvent fractions, the SCW *n*-BuOH and water fraction (SCWW)-treated groups showed significantly fewer writhes than the control group and the SCWW group had the highest antinociceptive effect among all fractions.

Therefore, effective compound isolation was performed using the SCWW sample and a Diaion HP-20 column. First, 0, 40 and 100% MeOH were used to obtain effective fractions. Then, seven fractions were extracted from the 40% MeOH isolated fraction. Among them, the fraction 4-treated mice group showed a significantly higher antinociceptive effect than the other mice groups in the acetic acid-induced writhing test. Fraction 4 was identified as 5-HMF.

5-HMF and its derivatives showed significantly fewer writhes in the acetic acid-induced writhing test, and less nociceptive behavior in the formalin-induced pain test (1st, and 2nd phase) at a dose of 10 mg/kg than the control group. Especially, 5-SMF presented a higher effect than the other groups; thus, 5-SMF was selected to evaluate the antinociceptive effect. 5-SMF showed greater effects than gabapentin in the acetic acid-induced writhing and 5% formalin tests. Furthermore, 5-SMF presented greater antinociceptive effects than gabapentin in the tail-flick test and hot-plate tests in the vincristine-induced neuropathic pain model. Therefore, 5-SMF may be an effective candidate for the alleviation of pain.

Based on the present study, we concluded that SCW is a functional food source associated with antinociceptive effects and that the synthesized 5-SMF compound is an effective therapeutic option for the treatment of neuropathic pain. Furthermore, this study has clinical significance in that it eliminates the toxicity of 5-HMF, which can cause disease, through synthesis, and confirm the antinociceptive effect of synthetic products.

## 4. Materials and Methods

### 4.1. Chemicals and Reagents

Methanol (MeOH), ethyl acetate (EtOAc) and butanol (*n*-BuOH) was acquired from J. T. Baker Chemical Company (Phillipsburg, NJ, USA) and all chemicals were ready to analytical grade.

### 4.2. Plant Materials

*Symplocos chinensis* f. pilosa Ohwi was collected from the Gangwon-do Forestry Development Research Institute (May, 2010) and air dried at room temperature for 5 days. A voucher specimen (No. 2010-RIC-2105) was deposited at Regional Innovation Center, Hallym University, ChunCheon, Korea.

### 4.3. Preparation of Extraction and Isolation

*Symplocos chinensis* f. pilosa Ohwi was subjected to extraction with water at room temperature. The filtrate was dried using freeze dryer (PVTFD10A, Ilshin, Seoul, Korea). The SCW was partitioned into three fractions: ethyl acetate (EtOAc), butanol (*n*-BuOH), and water. Among the fractions, the water fraction was purified using Diaion HP-20 column with MeOH solvent (0, 20, 40, 60, 80 and 100%). After that, 5-HMF was isolated from MeOH 40% fraction by using RP C_18_ column with gradient solvent system (H_2_O:MeOH = 9:1–1:1), and recrystallized.

### 4.4. Chemistry

#### 4.4.1. Synthesis of 5-Acetoxy Methyl Furfural (5-AMF) (**1**)

A suspension of 5-HMF (23.8 mM) in 2,2,2-trimethylpentane (Isooctane) (0.1 L) was refluxed (200 rpm) with lipase (2.8 g), molecular sieves (5 g) and acetic acid (119 mM) for 2 h at 60 °C; yellow lipid; ^1^H-NMR (CDCl3): δ (ppm) 9.576 (s, 1H), 7.504 (d, *J* = 2.1 Hz, 1H), 6.789 (d, *J* = 2.1 Hz, 1H), 5.131 (s, 2H), 2.077 (s, 3H); ^13^C-NMR: 178.3, 169.7, 155.2, 152.2, 123.6, 112.9, 57.4, 20.4; MS: 168 [M]^+^; IR: 1742, 1678, 1271, 1223, 1023 cm^−1^ (Figure 9).

#### 4.4.2. Synthesis of 5-Propionoxy Methyl Furfural (5-PMF) (**2**)

5-HMF (23.8 mM) was refluxed (200 rpm) in 2,2,2-trimethylpentane (Isooctane) (0.1 L) with lipase (2.8 g), molecular sieves (5 g) and propionic acid (47.6 mM) for 2 h at 60 °C; yellow lipid; ^1^H-NMR (CDCl3): δ (ppm) 9.563 (S, 1H), 7.368 (d, *J* = 3.61 Hz, 1H), 6.681 (d, *J* = 3.43 Hz, 1H), 5.035 (s, 2H), 2.355 (q, *J =* 7.58 Hz, 2H), 1.097 (t, *J* = 7.60 Hz, 3H); ^13^C-NMR: 175.9, 153.49, 151.66, 112.35, 110.8, 99.5, 59.3, 28.5, 9.7; MS: 181[M − H]^−^; IR: 1738, 1680, 1166, 1020 cm^−1^.

#### 4.4.3. Synthesis of 5-Butyroxy Methyl Furfural (5-BMF) (**3**)

5-HMF (23.8 mM), lipase (2.8 g), molecular sieves (5 g) and butyric acid (47.6 mM) was refluxed (200 rpm) in 2,2,2-trimethylpentane (Isooctane) (0.1 L) for 2 h at 60 °C; yellow lipid; ^1^H-NMR (CDCl3): δ (ppm) 9.468 (s, 1H), 7.272 (d, *J* = 3.56 Hz, 1H), 6.585 (d, *J* = 3.67 Hz, 1H), 4.942 (s, 2H), 2.200 (t, *J* = 7.30 Hz, 2H), 1.522 (sextet, 3H), 0.820 (t, *J* = 7.48 Hz, 2H); ^13^C-NMR: 173.7, 152.0, 150.2, 110.9, 109.42, 98.1, 57.8, 35.7, 18.4, 12.8; MS: 195 [M − H]^−^; IR: 1737, 1679, 1190, 1163, 1022 cm^−1^.

#### 4.4.4. Synthesis of 5-Succinoxy Methyl Furfural (5-SMF) (**4**)

A suspension of 5-HMF (8 mM) in 2,2,2-trimethylpentane (Isooctane) (0.033 L) was refluxed (200 rpm) with lipase (1.65 g), molecular sieves (1.65 g) and succinic acid (15.8 mM) for 2 h at 60 °C; white powder; ^1^H-NMR (CDCl3): δ (ppm) 9.563 (s, 1H), 7.365 (d, *J* = 3.58 Hz, 1H), 6.691 (d, *J* = 3.53 Hz, 1H), 5.173 (s, 2H), 4.880 (Br, s, 1H), 2.617 (m, 4H); ^13^C-NMR: 180.0, 176.5, 173,9, 157.8, 154.7, 124.3, 113.9, 59.4, 30.2, 29.8; MS: 227[M + H]^+^; IR: 1729, 1690, 1234, 1204, 939 cm^−1^.

#### 4.4.5. Synthesis of 5-Glutaroxy Methyl Furfural (5-GMF) (**5**)

5-HMF (8 mM), lipase (1.65 g), molecular sieves (1.65 g) and glutaric acid (15.8 mM) was refluxed (200 rpm) in 2,2,2-trimethylpentane (Isooctane) (0.033 L) for 2 h at 60 °C; yellow powder; ^1^H-NMR (CDCl3): δ (ppm) 9.562 (s, 1H), 7.368 (d, *J* = 3.56 Hz, 1H), 6.689 (d, *J* = 3.66 Hz, 1H), 5.165 (s, 2H), 4.948 (Br, s, 1H), 2.441 (t, *J* = 7.338 Hz, 2H), 2.346 (t, *J* = 7.369 Hz, 2H), 1.874 (m, 2H); ^13^C-NMR: 180.0, 177.2, 177.1, 154.7, 153.5, 114.0, 112.4, 59.3, 34.3, 34.1, 21.7; MS: 241 [M + H]^+^; IR: 1726, 1695, 1234, 1204, 1025, 919 cm^−1^.

#### 4.4.6. Synthesis of 5-Cinnamoxy Methyl Furfural (5-CMF) (**6**)

A suspension of 5-HMF (8 mM) in 2,2,2-trimethylpentane (Isooctane) (0.033 L) was refluxed (200 rpm) with lipase (1.65 g), molecular sieves (1.65 g) and vinyl-cinnamate (8 mM) for 2 h at 60 °C; yellow lipid; ^1^H-NMR (CDCl3): δ (ppm) 9.61, 7.64, 7.3, 7.21, 7.18, 7.14, 6.50, 6.39, 5.26; ^13^C-NMR: 178.0, 165.0, 159.0, 153.0, 141.3, 134.9, 128.4, 127.7, 126.2, 122.4, 119.4, 110.6, 67.5; MS: 257 [M + H]^+^; IR: 1727, 1695, 1235, 1204, 1025, 922 cm^−1^.

#### 4.4.7. Synthesis of 5-Lauroxy Methyl Furfural (5-LMF) (**7**)

5-HMF (1.8 mM) suspension in 2,2,2-trimethylpentane (Isooctane) (0.005 L) was refluxed (200 rpm) with lipase (0.14 g), molecular sieves (0.25 g) and lauric acid (8.98 mM) for 2 h at 60 °C; yellow powder; ^1^H-NMR (CDCl3): δ (ppm) 9.566 (s, 1H), 7.365 (d, *J* = 3.55 Hz, 1H), 6.678 (d, *J* = 3.61 Hz, 1H), 5.152 (s, 2H), 2.264 (t, *J* = 7.39 Hz, 2H), 1.587 (t, *J* = 7.20 Hz, 2H), 1.286 (s, 14H), 0.890 (s, 3H); ^13^C-NMR: 178.1, 175.3, 154.3, 151.68, 112.4, 110.8, 59.2, 35.4, 33.5, 31.2, 31.0, 30.9, 26.5, 24.2, 14.8; MS: 290 [M − OH_3_]^−^; IR: 1725, 1697, 945 cm^−1^.

### 4.5. Animals

ICR male mice (25–30 g, 6 weeks) were purchased from Koatech (Seoul, Korea). Animals were housed four per one cage in a room (22 ± 1 °C) with an alternating 12 h light-dark cycles. The animals were allowed to adapt at least 2 h before testing and were only use one time. For reducing variation, all experiments were performed during the light phase of the cycle (10:00–17:00). Food and water were available *ad libitum*. The experiment was approved by the Hallym University Institutional Animal Care and Use Committees (Registration Number: Hallym 2012-43), according to the ‘Guide for Care and Use of Laboratory Animals’ published by the National Institutes of Health and the ethical guidelines of the International Association for the Study of Pain. All mouse groups were euthanized after completion of experiments with 2,2,2-tribromoethanol (Sigma Aldrich, Saint Lopuis, MO, USA) and 2-methyl-2-butanol (Sigma Aldrich, Saint Louis, MO, USA). 

### 4.6. In Vivo Studies

#### 4.6.1. Induction of Neuropathic Pain Model

##### Chronic Constriction Injury (CCI) Neuropathic Pain Model

CCI model was induced as described in the previous method [28]. Mouse was anesthetized using 2,2,2-tribromoethanol. The skin of the lateral surface of left thigh was incised and cut directly through the biceps femoris muscle to expose the sciatic nerve. Chromic gut (4-0) were loosely tied around the nerve proximal part with a 1 mm distance. Double knots were used to prevent knot slippage. After performing surgery, muscular and skin layer were sutured with silk thread. Animals were housed individually after surgery.

##### Spared Nerve Injury (SNI) Neuropathic Pain Model

SNI model was performed under previous method [23]. The left hindlimb was immobilized and three peripheral branches (sural, common peroneal, and tibial nerves) exposed without damage. SNI surgery performed tight ligation of tibial and common peroneal nerves with a 5-0 silk, and removal of a 2 mm nerve portion. The third peripheral branch, the sural nerve, remains intact and any contact or stretch to this nerve is carefully avoided.

##### Vincristine Induced Neuropathic Pain Model

Peripheral neuropathy was induced in mice by administration of vincristine sulfate (100 mg/kg, i.p.) for seven consecutive days as described by Saika [29]. 

#### 4.6.2. Writhing Test

For the writhing test [30], 1% acetic acid was injection (i.p.) and then, the animals were immediately placed in an acrylic observation chamber (20 cm high, 20 cm diameter). The number of writhes was counted during 30 min after the injection of acetic acid. A writhe was defined as a contraction of the abdominal muscles accompanied by an extension of the forelimbs and elongation of the body.

#### 4.6.3. Formalin (5%) Test 

For the formalin test [31], 10 μL of 5% formalin was injected subcutaneously under the plantar surface of the left hind paw. Following injection of formalin, the animals were immediately placed in an acrylic observation chamber, and the time spent licking, shaking and biting the injected paw was measured with a stop-watch timer and considered as indication of nociception. The first phase of the nociceptive response normally peaked 0 to 5 min, and the second phase 20 to 40 min after formalin injection, representing the direct effect on nociceptors and inflammatory nociceptive responses, respectively [32].

#### 4.6.4. Tail-Flick Test

Antinociception was determined by the tail-flick [33]. For the measurement of the tail-flick latency, mice were gently held with one hand with the tail positioned in the apparatus (EMDIE Instrument Co., Maidens, VA, USA, Model TF6) and the tail-flick response was elicited by applying radiant heat to the dorsal surface of the tail. The intensity of radiant heat was adjusted so that the animal flicked its tail within 3 to 5 s.

### 4.7. Von Frey Filament Tests

For the von Frey test, the number of animals used in the experiment was 4 in each group. The tactile withdrawal threshold of mice was measured by using the von Frey method [34,35]. Mice were individually placed in a clear glass cells with a metal mesh floor allowed to adapt to the testing environment for 30 min. After an acclimation period, we stimulated the plantar surface of the left hind paw vertically with a series of von Frey filaments (North Coast Medical, Inc., Gilroy, CA, USA) with logarithmically increasing stiffness. The filament was bent for 5 s to the central plantar surface with a sufficient force, and brisk withdrawal or paw flinching was considered as a positive response. The test of tactile withdrawal threshold was repeated two times in each mouse, and the mean value was calculated. Mechanical hyperalgesia was assessed from 0 to 120 min after the administration of normal saline (5 mL/kg, b.w., p.o.), and samples. 

### 4.8. Bacterial Reverse Mutation (Ames) Test

Bacterial reverse mutation test was processed using Ames MPF TM 98/100 kit (Xenometrix, Basel, Switzerland). Study was conducted with testing strains of TA98, TA100, TA1535 and TA 1537.

### 4.9. Statistical Analysis

All values are expressed as the mean ± SD. Comparison between groups was performed using Student’s *t*-test. *p* < 0.05 was considered statistically significant.

## 5. Conclusions

In summary, the results of the present study demonstrated the antinociceptive effects of SCW and synthesized compound, 5-SMF, against neuropathic pain. Our data in an acetic acid-induced writhing test and a formalin-induced pain test suggest that SCW has antinociceptive activity on peripheral pain, and SCW showed a higher effect in the CCI and SNI models than the vehicle group. After evaluating the effect of SCW, the effect of solvent fractions and isolated compound from the SCW sample were confirmed. Among the fractions, water fraction was selected for isolation and 5-HMF was isolated. 5-HMF and its derivatives showed significantly effects in acetic acid-induced writhing test, formalin-induced pain test. In particular, 5-SMF presented a higher effect than the other groups in the neuropathic pain model. 

## Data Availability

Data are available upon request from the corresponding authors.

## Supplementary Materials

The following are available online: Figure S1: Chromatogram of the SCW and 5-hydroxymethylfural (5-HMF), Figure S2: ^1^H-NMR of 5-HMF, Figure S3: ^13^C-NMR of 5-HMF, Figure S4: HMBC of 5-HMF, Figure S5: EI-MS of 5-HMF.

## Sample Availability

Samples of the compounds are not available from the authors.
