# Supplementary material for: Effect of Extract and Synthesized Derivatives of Isolated Compound from Symplocos chinensis f. Pilosa Ohwi on Neuropathic Pain in Mice
Source: Molecules. 2021 Mar 15;26(6):1639. doi: 10.3390/molecules26061639 (PMC7999106; doi:10.3390/molecules26061639)
Supplement: Supplementary file 1 [file molecules-26-01639-s001.zip › molecules-1107322-supplementary.pdf]

molecules

MDPI

Supplementary Materials`

Effect of Extract and Synthesized Derivatives of Isolated Compound from *Symplocos Chinensis* f. Pilosa Ohwi on Neuropathic Pain in Mice.

Hyun-Yong Kim [1,†], Soo-Hyun Park [2,†], Guanglei Zuo [1], Kang Hyuk Kim [1], Seung Hwan Hwang [1,3], Hong-Won Suh [4] and Soon Sung Lim [1,5,6,*]

Materials and Methods

1. Phytochemical Analysis: High-Performance Liquid Chromatography (HPLC)

Chromatographic analysis of the compounds from the SCW was performed on an HPLC system (Agilent 1200 series system, CA, USA). The separations were carried out under gradient conditions using an Agilent (Santa Clara, CA, USA) Eclipse XDB-C18 column (4.6 µm × 15 cm, with 3.5 µm particle size) at 37 °C. The mobile phase composed water containing 0.1% formic acid (A) and 5% MeOH (B), according to the following elution program: 0-100 % B (0–30 min); 100-0 % B (30–31 min); and 0 % B (31–40 min). Peaks were detected using a UV detector at 254 nm. The flow rate was 0.8 ml/min, and the injection volume was 10 µL.

2. Structure identification

The structures of isolated compounds were elucidated by ^{1}H, ^{13}C and HMBC NMR (Bruker AV600, LabX, Mundelein, IL, USA), and EI-MS (JEOL JMS-700; JEOL Ltd., Tokyo, Japan).

Figure S1. Chromatogram of the SCW and 5-hydroxymethylfural (5-HMF).

Figure S2. ^{1}H-NMR of 5-HMF

Figure S3. ^{13}C-NMR of 5-HMF

Figure S4. HMBC of 5-HMF

Figure S5. EI-MS of 5-HMF

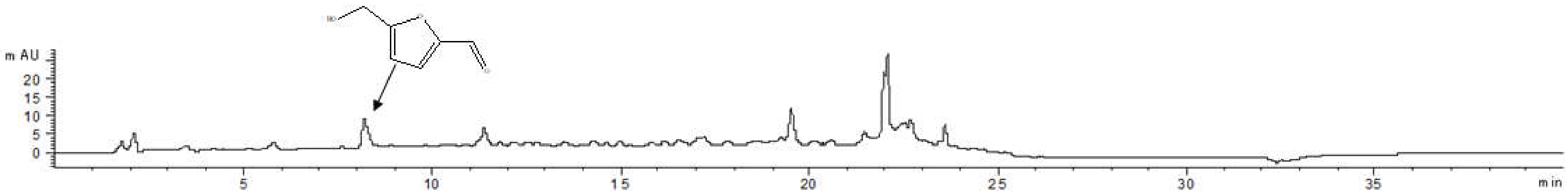

Figure S1. Chromatogram of the SCW and 5-hydroxymethylfural (5-HMF).

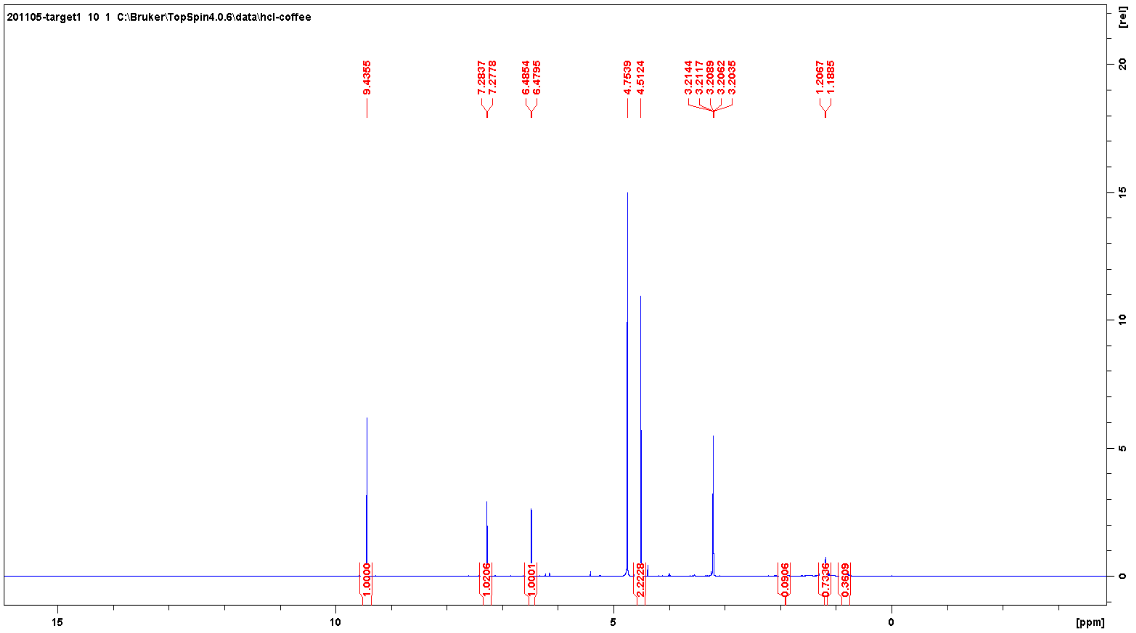

Figure S2. ^{1}H-NMR of 5-HMF

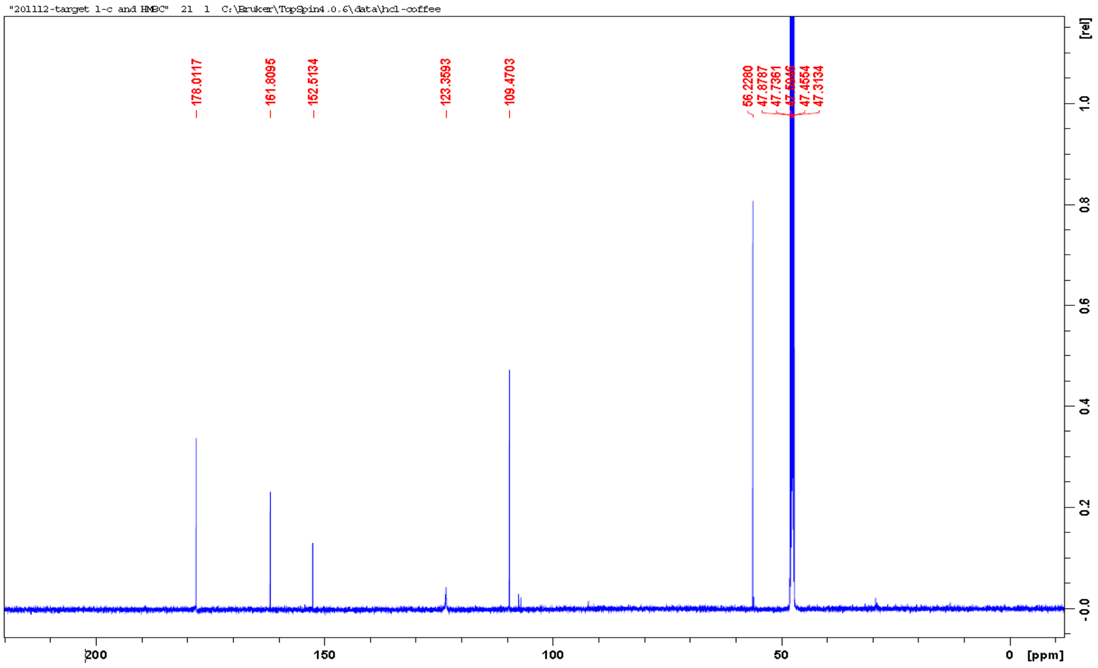

Figure S3. ^{13}C-NMR of 5-HMF

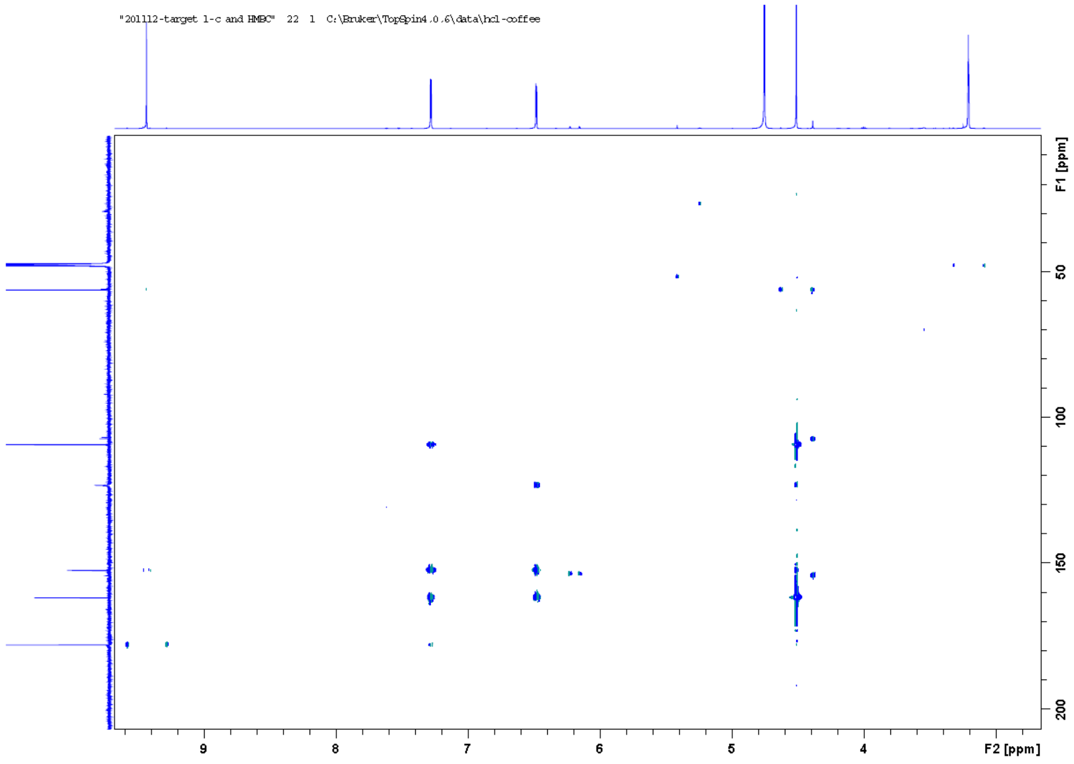

Figure S4. HMBC of 5-HMF

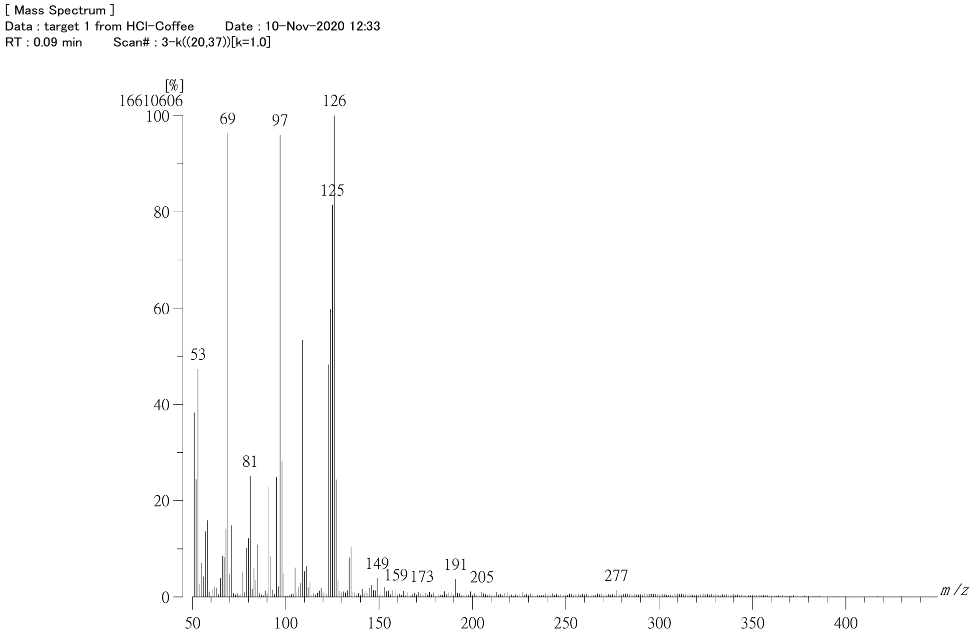

Figure S5. EI-MS of 5-HMF
